# COVID-19 vaccine hesitancy among indigenous people in Sudan: An incipient crisis

**DOI:** 10.1016/j.amsu.2022.103379

**Published:** 2022-02-15

**Authors:** Sarya Swed, Tasneem Mohamed, Rama Sakkour, Karam R. Motawea, Haidara Bohsas

**Affiliations:** a Faculty of Medicine, Aleppo University, Aleppo, Syria; b Neurosurgery Department/Ribat University, Hospital Burri, khartoum, Sudan; c Tishreen University, Latakia, Syria; d Alexandria University, Alexandria, Egypt

**Keywords:** Indigenous people, Sudan, COVID-19 vaccine, Vaccine hesitancy

Dear Editor,

In December 2019, a new coronavirus illness (COVID-19) caused by the severe acute respiratory syndrome coronavirus 2 (SARS-CoV-2) was discovered in Wuhan, Hubei Province, China [1].

Sudan is a low-income nation with a diversified ethnic mix, with a population of around 44 million people, according to the UN. Sudan was unprepared to handle and contain the COVID-19 epidemic that was looming.

COVID-19 was diagnosed slowly, using CT scans and PCR with reverse transcription (RT-PCR). On March 12, 2020, a 50-year-old Sudanese man came via plane from the United Arab Emirate with the first case of COVID-19. With positive COVID-19 RT-PCR testing, he had respiratory symptoms, was brought to the hospital, and died three days later. This heightened concerns about the arrival of SARS-CoV-2 in Sudan.

Since the outbreak began, Khartoum, the capital city, has been the heart of the pandemic, harboring more than 70% of all COVID-19 cases. Sudan had a total of 30,404 cases recorded on April 1, 2021, with 21,728 (72%) of those in Khartoum [2].

Cases have been documented in all 18 states, with Khartoum, Al Jazirah, and Gedaref among the worst-affected. Despite the fact that Khartoum State accounts for the bulk of the country's recorded cases, the majority of COVID-19-related deaths have been reported from areas outside than the capital [3].

Sudan has received 3.4 million doses of the AstraZeneca vaccine, according to UNICEF. According to government data, Sudan has barely gotten a quarter of the immunizations it requires.

The Sudanese government has vaccinated around 830,000 individuals out of a total population of 45 million since March. The coronavirus has caused more than 37,500 illnesses and 2800 fatalities in Sudan thus far. Given the rarity of testing, the genuine numbers are likely to be far higher [4].

The first doses of the vaccination were accessible in nine states, according to the Ministry of Health: Khartoum, Al-Jazeera, the Nile River, north-west Kordofan, south Darfur, North Darfur, North Kordofan, and the Red Sea [5].

Despite numerous interventions planned and implemented by the Federal Ministry of Health, with support from State Ministries of Health and partners such as UNICEF, the World Health Organization (WHO), and the World Bank, a few localities continue to have low vaccine uptake compared to the target populations, particularly among the elderly and those with illnesses [6].

Depending on published research, we find that there is a significant shortage of public awareness forward COVID-19 in Sudanese population [7].

UNICEF, GAVI, WHO, and other global humane organizations must perform suitable plans to reduce the disawareness to deal with the global pandemic and create cultural courses for increasing the attention to take COVID-19 vaccine for prevention [8].

While COVID-19 cases continue to be reported in Sudan, Ismail El Adani, Director of the Federal Ministry of Health's Immunization Department, stated that the AstraZeneca and Johnson vaccines are available in all states, while the Sinopharm vaccine is only available in the states of El Gezira and Khartoum [Fig. 1]. UNICEF is collaborating with the Sudanese government, WHO, COVAX, and other partners to provide more COVID-19 vaccine doses in the coming quarter in order to reach a larger number of individuals before the end of the year [9].

**Fig. 1:**
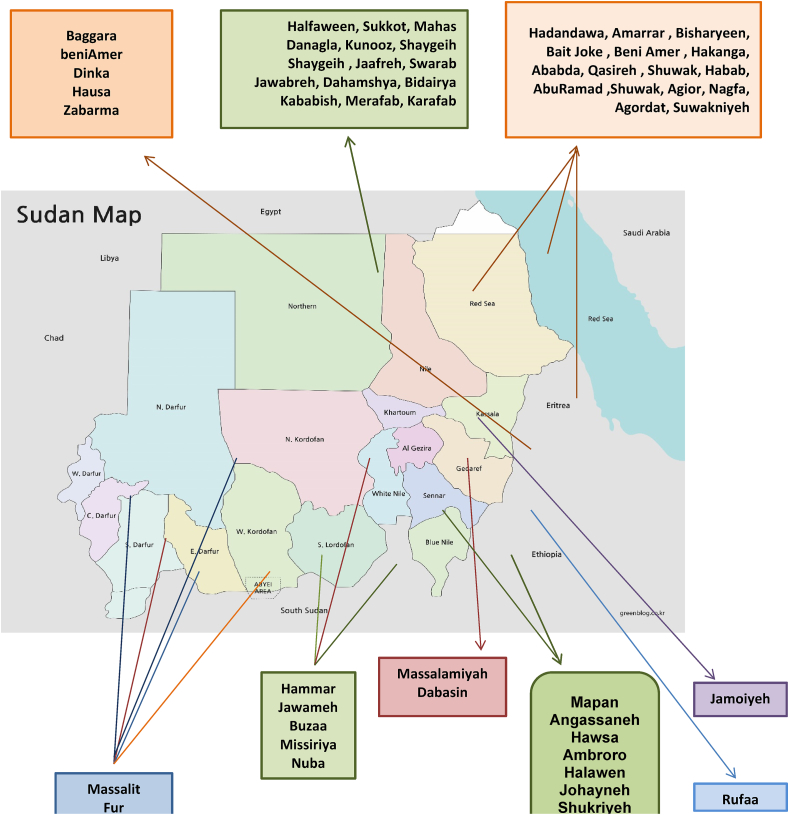
THE Map of Sudan that shows the distribution of neglected tribes that do not receive vaccination_COVID19.

As of October 20, 2021, Sudan had delivered at least 1,659,666 doses of COVID vaccination. Sudan received an average of 5151 doses per day. It will take another 1663 days to give enough dosages for another 10% of the population at this rate [10]. Even though these reported statistics, the remaining shortage of COVID-19 vaccines create a significant problem at all levels in Sudanese public health in addition to the severe ignorance in many rural regions forward the importance of receiving COVID-19 vaccines and following the preventive procedures. The key stakeholders should continue to build support for the GAVI vaccine alliance and the Coalition for Epidemic Preparedness Innovations (COVAX) global vaccines initiative, which aims to provide two billion vaccine doses to 92 middle and low-income governments through December 2021 [11].

Unfortunately, these health problems related to COVID-19 are distributed in many Arabic low-income countries such as Syria [12], Yemen [13] and Somalia.

To address COVID-19, responsible authorities should organize awareness initiatives and adequate healthcare facilities, particularly for indigenous peoples in remote regions.

## Provenance and peer review

Not commissioned externally peer reviewed.

## Sources of funding

This research did not receive any specific grant from funding agencies in the public, commercial, or not-for-profit sectors.

## Ethical approval

N/A.

## Consent

N/A.

## Author contribution

All authors have participated in writing and reviewing the paper.

## Registration of research studies

Not applicable.

## Guarantor

Sarya Swed.

## Declaration of competing interest

All authors declare no conflict of interest.
